# Nanomedicine against Aβ Aggregation by β–Sheet Breaker Peptide Delivery: In Vitro Evidence

**DOI:** 10.3390/pharmaceutics11110572

**Published:** 2019-11-01

**Authors:** Francesca Pederzoli, Barbara Ruozi, Jason Duskey, Simone Hagmeyer, Ann Katrin Sauer, Stefanie Grabrucker, Romina Coelho, Natalia Oddone, Ilaria Ottonelli, Eleonora Daini, Michele Zoli, Maria Angela Vandelli, Giovanni Tosi, Andreas M. Grabrucker

**Affiliations:** 1 Department of Life Sciences Te.Far.T.I. Research Center, University of Modena and Reggio Emilia, 41124 Modena, Italy; francesca.pederzoli@unimore.it (F.P.); barbara.ruozi@unimore.it (B.R.); jasonthomas.duskey@unimore.it (J.D.); natoddone@gmail.com (N.O.); ilaria.ottonelli@gmail.it (I.O.); mariaangela.vandelli@unimore.it (M.A.V.); 2 Cellular Neurobiology and Neuro-Nanotechnology Lab, Department of Biological Sciences, University of Limerick, Limerick V94PH61, Ireland; simone.hagmeyer@ul.ie (S.H.); Ann.Katrin.Sauer@ul.ie (A.K.S.); stefanie.grabrucker@ul.ie (S.G.); 3 Biosystems and Integrative Sciences Institute, Faculdade de Ciências, Departamento de Química e Bioquímica, Universidade de Lisboa, 1749-016 Lisbon, Portugal; rjccoelho@fc.ul.pt; 4 Department of Biomedical, Metabolic and Neural Sciences, Center for Neuroscience and Neurotechnology, University of Modena and Reggio Emilia, 41124 Modena, Italy; eleonora.daini@unimore.it (E.D.); mzoli@unimore.it (M.Z.); 5 Bernal Institute, University of Limerick, Limerick V94T9PX, Ireland; 6 Health Research Institute (HRI), University of Limerick, Limerick V94T9PX, Ireland; 7 Synthesis and Solid State Pharmaceutical Centre, University of Limerick, Limerick V94T9PX, Ireland

**Keywords:** Alzheimer disease (AD), Polymeric nanoparticles (NPs), KLVFF peptide, blood-brain barrier (BBB), amyloid β, Aβ

## Abstract

The accumulation of amyloid β (Aβ) triggers a cascade of toxic events in Alzheimer’s disease (AD). The KLVFF peptide can interfere with Aβ aggregation. However, the peptide suffers from poor bioavailability and the inability to cross the blood–brain barrier. In this work, we study the possibility of adopting nanomedicine to overcome KLVFF limits in biodistribution. We produced new engineered polymeric nanoparticles (NPs), and we evaluated the cellular toxicity of these NPs and validated that KVLFF peptides released by NPs show the same promising effects on AD pathology. Our results revealed the successful generation of KVLFF loaded NPs that, without significant effects on cell heath, are even more potent in reversing Aβ-induced pathologies compared to the free peptide. Therefore, NPs will significantly advance KVLFF treatment as a therapeutic option for AD.

## 1. Introduction

Alzheimer’s disease (AD) is characterized by the abnormal extracellular accumulation of the amyloid-beta (Aβ) protein and/or its assembly into paired helical filaments and plaques. The progressive accumulation of Aβ aggregates is widely believed to be fundamental to the initial development of the neurodegenerative pathology and to trigger neurotoxicity, oxidative damage, and inflammation that contribute to the progression of AD [1]. Recently, several strategies were investigated, aiming to influence the Aβ peptide aggregation pathway in order to either favor its elimination and, therefore, the balance between production and clearance, or to slow the aggregation process [2,3,4]. Elucidation of Aβ fibril structural properties enabled the design and discovery of drugs that specifically interfere with Aβ–Aβ interaction and polymerization, thereby inhibiting the formation of fibrils or dissolving preformed fibrillar aggregates of Aβ. 

Amongst these different drugs, a pentapeptide (Lys-Leu-Val-Phe-Phe, named KLVFF) specifically designed to target a central region of Aβ was confirmed to inhibit the elongation of fibrillar aggregates in vitro [5,6], by interacting with a homologous sequence on the oligomeric form of fragment Aβ-42 [6]. Both electrostatic forces and hydrogen bonds lead to the formation of a link between the pentapeptide and the Aβ-42 fragment, causing a change in the secondary structure to an antiparallel β-sheet formation [7]. This conformation interferes with the amyloid plaque formation process by reducing aggregation and non-toxic minor fibril formation [8,9,10], and more importantly, KLVFF can disaggregate fibrils by binding to hydrophobic regions of Aβ [11,12]. Unfortunately, the KLVFF peptide displays poor aqueous solubility, low stability, slight cellular uptake, and the inability to cross the blood–brain barrier (BBB). To increase KLVFF’ s stability, several modifications in the peptide structure, such as methylation [13] or the addition of an amino acid such as proline [14], were tested, but the poor properties still hamper in vivo applications. 

To overcome the limitations, a powerful approach to improve the pharmacokinetic profile of drugs is based on the exploitation of polymeric nanocarriers. Very recent experiments described the use of a nanocomposite featured by small-sized particles (lower than 20 nm) with KLVFF integrated on the surface [15], with interesting readouts in terms of changes in Aβ aggregate morphology and a decrease in neuron damage in vitro and in AD mice, confirming the possible role of KLVFF to remarkably impact on the Aβ aggregation process.

The simultaneous use of both peptides and polymers was also positively applied by recent findings, describing the production and testing of self-destructive nanosweepers based on multifunctional peptide-polymers, such as chitosan and polyethylene glycol (PEG) [16], or poly (hydroxypropyl methacrylamide) [17], able to capture and clear Aβ for the effective treatment of AD.

All of these approaches aim to stabilize peptides, but may be limited by the nature of the polymer used in terms of impact on slowing down the rapid metabolism and systemic elimination or modulating the peptide’s release. In this view, the use of polymeric nanoparticles (NPs) can increase the water solubility, improving drug biodistribution and bioavailability. Amongst the different materials, few polymers guarantee the safety profiles; thus, given its favorable biocompatibility and biodegradability properties, poly(d,l-lactide-*co*-glycolide) (PLGA) is one of the most successful polymers used in the development of drug delivery systems, particularly in brain targeting [18], and is widely used for drug delivery across the BBB by means of tailored PLGA NPs. This has been proven in several in vivo studies using relevant pathological models [19,20,21,22]. Thus, application of this drug delivery technology could indeed be a promising option for the in vivo treatment of AD.

The poor solubility of KLVFF in water-based phases (buffer or surfactant solution) negatively affects the routinely proposed methods of nanoparticle preparation to load peptides (for example, double emulsion technology), by inhibiting encapsulation efficiency. Thus, in this research, we developed a novel biocompatible nanomedicine-based carrier able to control the release and stabilize KLVFF. Nanocarriers were prepared by adapting the nanoprecipitation procedure using an unusual mixture of organic solvents (Dimethyl sulfoxide (DMSO)/Acetone) to dissolve the polymer and peptide, forming stable loaded NPs. The effect of released KLVFF by NPs in inhibiting the amyloid formation or in inducing disaggregation of formed or in-formation plaques was then tested in in vitro models.

## 2. Materials and Methods 

### 2.1. Materials

Poly(d,l-lactide-*co*-glycolide) acid (PLGA RG-503H 50:50, inherent viscosity in 0.1% (*w*/*v*) CHCl_3_ at 25 °C = 0.38 dL/g) was used as received from the manufacturer (Boehringer-Ingelheim, Ingelheim am Rhein, Germany). According to the experimental titration results of the carboxylic end of the polymers (4.94 mg KOH/g polymer), the molecular weight of RG-503H was calculated to be 11,000 Da. Peptide KLVFF (Lys-Leu-Val-Phe-Phe, C42H63N9O8, MW 822.01) was obtained from Mimotopes (Springvale Rd Mulgrave, Victoria, Australia). Pluronic F68 (molecular weight of 8500–9000 Da) was purchased from Sigma-Aldrich. Primary antibodies were purchased from Synaptic Systems (Homer1) and Abcam (MAP2). Fetal bovine serum (FBS) and phosphate-buffered saline (PBS) were purchased from Euroclone Celbio (Milan, Italy). Secondary Alexa Fluor conjugated antibodies were purchased from Invitrogen. Thioflavin was obtained from Sigma Aldrich. Synthetic Aβ peptide, human Aβ_(1–42)_, was purchased from Abcam. All the solvents were of analytical grade, and all other chemicals and media were used as received from the manufacturers, and unless otherwise indicated, obtained from Sigma-Aldrich.

### 2.2. Preparation of KLVFF-Loaded NPs

KLVFF-loaded NPs (K-NPs) were obtained with an optimized nanoprecipitation procedure. Preliminary KLVFF, PLGA, and KLVFF/PLGA-mix solubility tests were carried out in water and diffusible organic solvent (see Supplementary Table S1). K-NPs were prepared by solubilizing a fixed amount of KLVFF (3 mg) into DMSO (0.3 mL) and adding it to a heated (50 °C) acetone solution (3.7 mL) of PLGA RG-503H (50 mg). This hot organic mixture was added dropwise into a 3% (*w*/*v*) Pluronic F68 aqueous solution (12.5 mL). The solution was then stirred at room temperature (r.t.) for 15 min, and the organic solvent was removed at 30 °C under reduced pressure (10 mm Hg, Buchi Rotavapor R-300 Evaporation Systems, Flawil, Switzerland). The same procedure was applied to produce control NPs (CNT-NPs), with the unique difference of using a solution of DMSO without KLVFF. NP batches were then purified by centrifugation (16,000 rpm for 10 min, 4 °C; J2-21 model, Beckman centrifuge, NewPort Pagnell, UK), washed and re-suspended in water (4 mL). Samples were stored at −20 °C before use, or freeze-dried (LyoLab 3000, Heto-Holten, Allerod, Denmark) to allow weight yield evaluation. 

### 2.3. Characterization of NPs

The average diameter (Z-average), the size distribution (expressed as D(50) and D(90)) and the polydispersity index (PDI) of the NP samples were assessed by Photon Correlation Spectroscopy (PCS) using a Zetasizer Nano ZS (Malvern, Malvern, UK; Laser 4 mW He–Ne, 633 nm, laser attenuator automatic, transmission 100–0.0003%, detector avalanche photodiode, quantum efficiency (QE) >50% at 633 nm, at 25 °C). For each formulation, the mean diameter and PDI were calculated from three replicates of three different batches (nine measurements). The zeta potential (z-potential) (average of 10 measurements) was analyzed using the same equipment, with a combination of laser Doppler velocimetry and phase analysis light scattering (PALS). The Atomic Force Microscope (AFM) measurements were performed with an AFM (Park Instruments, Sunnyvale, CA, USA) operating at about 20 °C in air and in non-contact mode using a commercial silicon tip-cantilever (high resolution noncontact “GOLDEN” Silicon Cantilevers NSG-11, NT-MDT, tip diameter 5–10 nm; Zelenograd, Moscow, Russia) with a stiffness of about 40 mN and a resonance frequency around 150 kHz. After the purification, the NP sample was dispersed in distilled water before being applied on a freshly cleaved mica disk (1 cm × 1 cm); after 2 min, the excess water was removed using filter paper. The AFM images (a topographical image, and a second one showing the “error signal”) were obtained with a scan rate of 1 Hz. The error signal was obtained by comparing the image representing the amplitude of the vibrations of the cantilever with the amplitude of a reference point. The images obtained by this method showed small superficial variations of the samples. Images were processed using ProScan Data Acquisition software.

### 2.4. Evaluation of the Weight Yield

Freeze-dried samples were used to calculate weight recovery. In particular, the weight yield (WY%) was calculated as follows:

WY% = [mg of NPs recovered/(mg of PLGA + mg of peptide used for the preparation)] × 100.

### 2.5. Extraction of KLVFF from NPs

Briefly, a weight amount of NPs (about 5 mg) was solubilized into 1 mL of dichloromethane (DCM) by using a bath sonicator (Bransonic® bath Ultrasonic, Optolab Instrument, Modena, Italy) for 2 min; 1 mL of 1% (*w*/*v*) Polyvinyl Alcohol (PVA) solution was then added to extract the peptide. The mixture was shaken and then centrifuged (Spectrafuge 24D) to separate the two phases. The aqueous phase was collected, and the organic phase was again subjected to two further extractions. 

### 2.6. Quantification of KLVFF

Quantification of the peptide was obtained by using HPLC and a colorimetric assay with ninhydrin. In detail, using an HPLC instrument (JASCO Europe, Cremella, Italy) with a Model PU2089 pump and an injection valve with a 50 µL sample loop (Model 7725i, Jasco), and a UV/VIS detector (UV1575, Jasco), the freshly prepared solutions and mobile phases were filtered through a 0.45 µm hydrophilic polypropylene (PP) membrane filter (Sartorius, Goettingen, Germany). A C8 column (Aeris Widepure; 150 × 4.60 mm) equipped with a security guard was used as an analytical column. Elution was obtained using gradient steps of solvents A (0.1% trifluoroacetic acid in MilliQ water (pH 2.5)) and B (0.1% trifluoroacetic acid in acetonitrile) starting from 80:20 (A:B) to 20:80 in 13 min at a flow rate of 1.2 mL/min. After the run was complete, the column was re-equilibrated for 2 min. All analyses were carried out under isothermal conditions at 70 °C (Column Heater, model 7971, Jones Chromatography). The chromatographic peak area of the samples was recorded and analyzed using a JASCO software (ChromNav 2.0-Chromatography Data System program, Jasco). The concentration was calculated using a calibration curve (linearity was assumed in the range 2–38 µg/mL; *R*^2^ = 0.9994).

Ninhydrin assay: 100 μL of the sample (extracted from NPs) was added, in duplicates, in the wells of a 96-well plate with 75 μL of ninhydrin color reagent. The plate was then incubated at 80 °C for 30 min. Subsequently, the plate was cooled to r.t., and 100 μL of stabilizing solution (50% *v*/*v* ethanol) was added to each well. Finally, the spectrophotometric reading at 570 nm was performed through a Microplate Reader Multiscan (Spectrum Finstruments®). A standard concentration of peptide 1% *w*/*v* of PVA aqueous solution was used to generate a calibration curve. Linearity was assumed in the range of 2–35 µg/mL (*R*^2^ = 0.9979). All the data are shown as the mean of at least three measurements.

### 2.7. Encapsulation Efficiency and Loading Capacity 

The amount of KLVFF loaded into NPs was calculated as the percentage of loading capacity (LC) and encapsulation efficiency (EE) using the following equations:

LC = *D*/*W* × 100,

EE = *D*/*T*_d_ × 100,

where *D* is the amount of the encapsulated drug, *W* is the weight of NPs (polymer + drug), and *T*_d_ is the amount of drug added for the preparation. All data are expressed as the means of at least three determinations.

### 2.8. In Vitro Release Studies

For each time point, an exact amount of K-NPs (about 10 mg, KLVFF content about 0.1 mg) was suspended in 1 mL FBS:PBS (50:50 *v*/*v*) at 37 °C under magnetic stirring. After an incubation time (10 min, 1, 3, 24, 48, or 72 h), the suspension was centrifuged (13,000 rpm for 10 min) to separate K-NPs from the supernatant. Serum proteins that may be adsorbed on the NP surface were quantified using a colorimetric bicinchoninic acid (BCA) assay (Micro BCA protein assay kit composed of reagent A (alkaline tartrate–carbonate buffer), reagent B (bicinchoninic acid solution), and reagent C (copper sulfate solution)—Thermo Fisher Scientific Inc., Milan, Italy). The amount of adsorbed proteins was subtracted from the final value, representing the amount of plain NPs recovered after centrifugation. The KLVFF amount at each incubation time was determined after extraction from the NPs, as described above. The percentage of drug released was calculated using the following formula:

% of KLVFF released = (KLVFF tot − KLVFF residual) × 100,

where KLVFF tot is the initial drug content in the nanoparticle sample (*t* = 0), and the KLVFF residual is the amount of drug in the same sample after the incubation period.

### 2.9. Cell Culture 

Primary hippocampal cultures were prepared from rat hippocampi (BrainBits, Loughborough, UK). In brief, hippocampi were washed and transferred to 1800 μL HBSS (Hank’s Balanced Salt Solution, Sigma Aldrich) and then trypsinized with 200 μL Trypsin 2.5% for 20 min at 37 °C. After washing the cells five times with HBSS, they were re-suspended in 1600 μL HBSS with 400 μL DNAse 1 (0.01%). The suspension was then filtered through a 125 μm sieve and incubated with 18 mL of DMEM (Dulbecco’s Modified Eagle Medium, Sigma Aldrich, Arklow, Ireland) containing 10% Fetal Calf Serum (FCS), 1% glutamine, and 1% penicillin-streptomycin (DMEM+++). After cell-counting using a Neubauer chamber, the cells were seeded on poly-l-lysine (PLL) (0.1 mg/mL) coated glass coverslips in a 24-well plate at a density of 30,000 cells/well. After 24 h, the medium was changed to Neurobasal medium (Life Technologies via Biosciences, Dun Laoghaire, Ireland), complemented with B27 supplement (Life Technologies), 0.5 mM l-Glutamine (Life Technologies), and 100 U/mL penicillin/streptomycin (Life Technologies) (NB+++) and maintained at 37 °C in 5% CO_2_. 

### 2.10. Treatment of Hippocampal Cells 

To investigate the two possible impacts of treatment with KLVFF (inhibition of aggregation, and disaggregation and inhibition of re-aggregation), we set up two different experiments: to clarify the potency of inhibition of KLVFF on plaque formation (“inhibition test”), Aβ_(1–42)_ peptides (2 M) and KLVFF peptides (10 or 100 M) (or corresponding dose of KLVFF delivered by KLVFF-loaded NPs) were co-administered at time 0 and the experiments were performed over 24 h. 

To evaluate the effect of KLVFF on the disaggregation of formed A aggregates and inhibition of re-aggregation (“disaggregation test”), Aβ_(1–42)_ peptides (2 M) were administered to cells at time 0 and after 24 h, free KLVFF peptides (10 or 100 M) (or corresponding doses of KLVFF delivered by KLVFF-loaded NPs) were administered to cells.

To generate 200 μM (mainly monomeric) Aβ stock solution, lyophilized Aβ_(1–42)_ was dissolved in DMSO, vortexed for 30 min at RT, and centrifuged for 1 h (15,000× *g*) at 4 °C. The supernatant was aliquoted, snap-frozen, and stored at −20 °C. Further dilutions of the peptides were performed in the culture medium (NB+++). For the treatments, neurons were seeded in a total volume of 500 μL per well (24-well plate). Freeze-dried NP samples were re-suspended in HBSS under sonication (Emmi-05P Sonicator, Emag, Germany) to have a starting batch for further dilutions in NB+++. All the NP stock solutions were prepared according to the calculated KLVFF content, and the volume of HBSS was adjusted accordingly. Stock solutions of CNT-NPs were prepared to match the amount of NPs per mL of K-NPs. After the incubation period, cells were analyzed using immunocytochemistry and cell health assays.

### 2.11. Immunocytochemistry (ICC)

For ICC, the cells were fixed with 4% paraformaldehyde (PFA)/4% sucrose/PBS at 4 °C for 20 min. After washing three times for 5 min with 1× PBS containing 0.2% Triton X-100 at r.t., blocking was performed using a blocking solution (BS) containing 10% FBS in 1x PBS for 1 h at r.t. Then, samples were incubated with the primary antibodies (Homer1, MAP2) in BS at r.t. for 2 h or at 4 °C overnight, followed by three 5 min washing-steps with 1× PBS and incubation with the secondary antibody coupled with Alexa488, Alexa568, or Alexa647 in BS for 1 h. Cell nuclei were counterstained with DAPI (4′,6-diamidino-2-phenylindole). Finally, coverslips were mounted using Vecta Mount (Vector Laboratories, Burlingame, CA, USA). Aβ aggregates were visualized using Thioflavin T.

### 2.12. Cell Health Assay

The cell health of the neuronal cultures after treatment was measured using the healthy/apoptotic/necrotic cell detection kit (Promokine) according to the manufacturer’s protocol. In brief, cells were treated with 1× binding buffer and then incubated for 15 min at r.t. protected from light with a staining solution containing 100 μL of 1× binding buffer, 5 μL of FITC-Annexin V, 5 μL of Ethidium Homodimer III, and 5 μL of Hoechst 33342. Subsequently, cells were washed twice with the 1× binding buffer. Finally, the cells were fixed with (PFA)/PBS and 1.25 mM CaCl2 at r.t. for 15 min and coverslips mounted using Vecta Mount. As a positive control, apoptosis was induced with 500 μL of 70% ethanol. Fluorescence images were obtained with an inverted confocal LSM710 microscope (Zeiss) and ZEN software v.2.5. Quantification was performed using ImageJ 1.49j.

Alternatively, cells were seeded at a density of 5,000 cells per well on PLL-coated E-Plate 16 plate (ACEA Biosciences, San Diego, CA, USA) and treated with 2, 5, or 10 µM Aβ_(1–42)_ peptide after 24 h of growth. Controls were treated with vehicle (DMSO) only. Impedance was measured every 5 min in the following 36 h of treatment using the xCELLigence RTCA Systems. A decrease in impedance is associated with a decreased proliferation and/or the detachment of cells, and therefore a sign impaired cell health. Data are shown as slope, calculated according to the RTCA DPlus Software Manual V1.0.

### 2.13. Quantitative Real-Time PCR (qRT-PCR)

Isolation of total RNA was performed using the RNeasy Mini kit (Qiagen, Manchester, UK) according to the manufacturer’s instructions. First-strand synthesis and real-time qRT-PCR amplification (Roche LightCycler 480 II) were carried out in a one-step, single-tube format using the Rotor-Gene SYBR Green RT-PCR kit (Qiagen), and using validated primer pairs from Qiagen (Quantitect primer assay). The hydroxymethylbilane synthase (HMBS) gene was used as an internal standard. All reactions were run at least in technical triplicates, and mean ct values were transformed into virtual mRNA levels according to the formula: virtual mRNA level = 10 × ((ct_(target)_ – ct_(standard)_)/slope of the standard curve).

### 2.14. Statistics 

Statistical analysis was performed with SPSS version 20. All data are shown as mean ± SEM (standard error of the mean), if not indicated otherwise. For comparisons, one-way analysis of variance (ANOVA) was performed, followed by post hoc tests for within-group comparisons (Tukey test). Statistically significant differences are indicated in the figures by * *p* ≤ 0.05, ** *p* ≤ 0.01, and *** *p* ≤ 0.001.

## 3. Results

### 3.1. NPs Were Able to Load KLVFF

**Figure 1:**
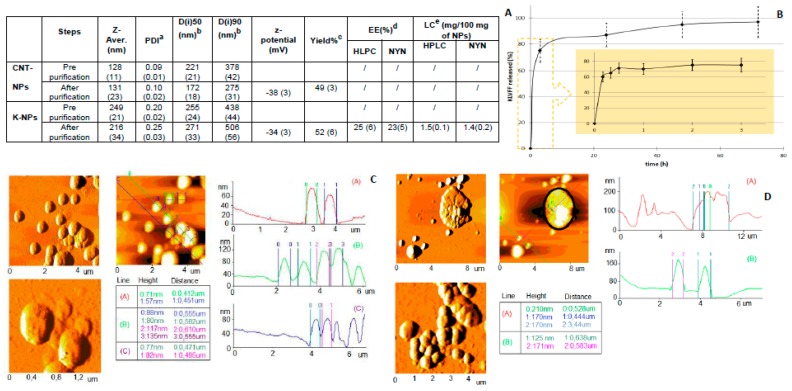
(**A**) Physico–chemical properties and technological characterization of samples. Values are mean ± SD (standard deviation) *(n* = 9) reported in brackets. (**B**) KLVFF release from NPs, (**C**) AFM analyses of CNT-NPs and (**D**) K-NPs. ^a^ polydispersity Index (PDI); ^b^ The results were expressed as intensity distribution (D(i)), i.e., the size below which is placed the 50% (D(i)50)) and 90% (D(i)90)) of all the particles; ^c^ the percentage of yield was expressed as the ratio of the recovered freeze-dried sample out of the total mass-weighted (polymer + drug) percent; ^d^ the percentage of encapsulation efficiency was expressed as the ratio of the encapsulated out of the total (encapsulated + free) drug percentage; ^e^ the drug content (loading capacity, LC) was expressed as the amount of the encapsulated drug in 100 mg of NPs.

The chemico–physical characteristics of the samples (CNT-NPs and K-NPs) are reported in Figure 1A. Unloaded CNT-NPs appeared to be stably formed, with a homogeneous size distribution (polydispersity index, PDI < 0.2), hydrodynamic diameter close to 130 nm, and were easily re-suspendable after purification. AFM images confirmed the homogeneity of CNT-NPs, as featured by distinct compact spherical structures with smooth surfaces (Figure 1C). 

The mean diameter of the NPs showed a slight increase when formulated with KLVFF (K-NPs). K-NPs showed a higher size (200 to 250 nm) and broader size distribution (PDI > 0.2). The increase in both Di (90) values and polydispersion indices of K-NPs indicated a marked higher sample-complexity, providing some indication of successful drug loading (Figure 1A).

The AFM analysis of K-NPs (Figure 1D) confirmed the presence of heterogeneous structures, characterized by a complex morphology and irregular shape, in which tangled and more agglomerate structures were observed. No correlation between PCS data and corresponding dimensional data was obtained by processing AFM images. Both samples on mica consisted of particles in which the heights did not correlate with diameters: this deformation could be due to the interaction between the sample and the substrate, as well as the continuous movement of the tip on the sample [23]. No differences in z-potential were observed with all NPs featured by negatively charged surfaces. 

The EE% was calculated to be approximately 25%, corresponding to about 1.5 mg of peptide per 100 mg of NPs (Figure 1A). After incubation in a simulated physiological environment (PBS with added FBS at 37 °C), more than 70% of the drug was released within the first 3 h (Figure 1B), while the portion of KLVFF stably loaded into the inner part of K-NPs slowly diffused, reaching a plateau after 72 h.

### 3.2. KLVFF Peptide Shows Beneficial Effects in an In Vitro Model for AD

**Figure 2:**
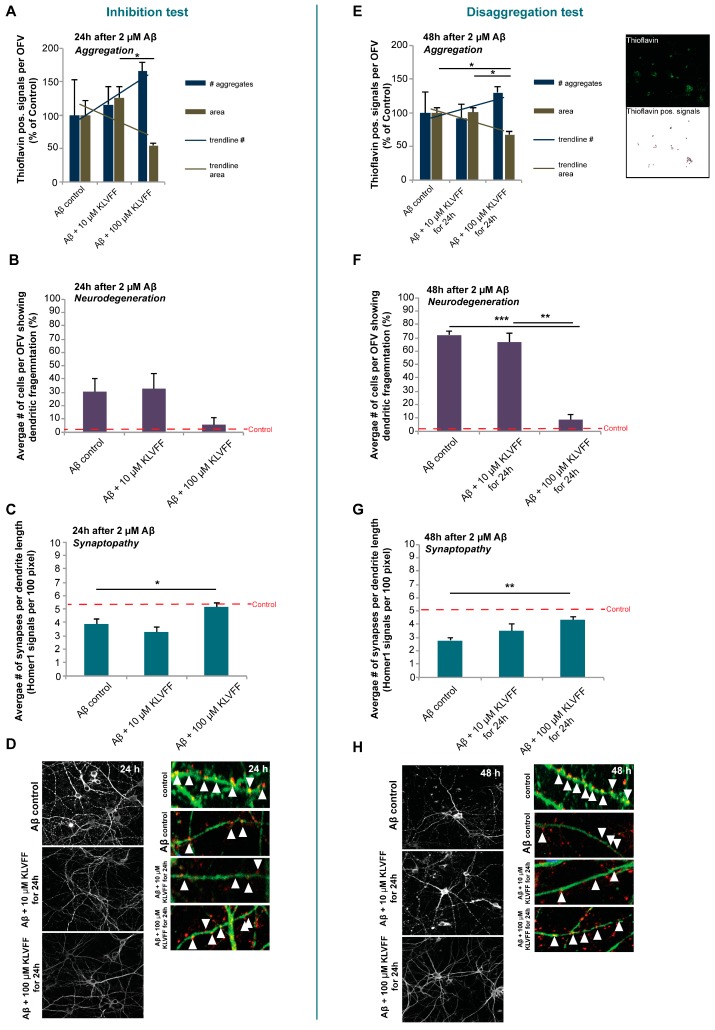
(**A**,**E**) The number and area (in pixel^2^) of aggregates visualized by Thioflavin staining and measured in three optic fields of view (OFV). The results are shown in percentage of control. (**B**,**F**) The number of cells showing fragmented dendrites (microtubule-associated protein 2 (MAP2) staining) was analyzed. (**C**,**G**) A “synaptopathy” was evaluated by measuring the number of Homer1 positive signals per dendrite length. Three optic fields of view (OFV) with at least ten cells were evaluated. (**D**) Left panel: MAP2 staining showing dendritic fragmentation as an early sign of neurodegeneration. Right panel: MAP2 (green) and Homer1 (red) staining visualizing synapses along a dendrite. (**H**) Left panel: MAP2 staining showing dendritic fragmentation as an early sign of neurodegeneration. Right panel: MAP2 (green) and Homer1 (red) staining visualizing synapses along a dendrite. A more detailed caption with the relevant statistical analysis data is reported in the supplementary material.

After K-NPs production, the incubation time and concentration-dependent effects of pure KLVFF peptide on rat hippocampal neurons exposed to human Aβ_(1–42)_ peptide were evaluated. As a first aim, inhibition tests were performed by treating at the same time (time 0) neurons with Aβ_(1–42)_ peptide (2 μM) and with two different doses of KLVFF peptide (10 μM or 100 μM). The minimal concentration of Aβ_(1–42)_ peptide that was detected previously to produce biological effects (Figure S1A,B) if monomeric Aβ_(1–42)_ (Figure S1C) is applied was chosen. Our results show that, even if it is possible to recognize a trend reduction of the area of Aβ_(1–42)_ aggregates produced in cell culture over an incubation time of 24 h when using high dose of KLVFF (100 M), no significant effect of both doses of KLVFF compared to controls treated with Aβ_(1–42)_ only was detected. The number of aggregates slightly, but non-significantly increased (Figure 2A). Therefore, at these doses, although a trend towards more but smaller aggregates can be seen, an apparent effect on the inhibition of Aβ aggregation was not observed.

Next, we evaluated whether the alteration in aggregation status had a possible effect on the neuronal pathology induced by Aβ_(1–42)_. To that end, neurons were labeled using microtubule-associated protein 2 (MAP2) as a marker to visualize dendritic morphology (Figure 2B,D), and Homer1 as a marker for excitatory synapses (Figure 2C,D). Concerning healthy cells (red dashed line in any panel), positive control samples (24 h of treatment with only Aβ_(1–42)_) highlighted that about 30% of dendrites showed signs of fragmentation (Figure 2B,D) and also a relevant decrease in synapses (Figure 2C,D). Inhibition tests indicated that the dose of 10 μM KLVFF co-administered at time 0 with Aβ_(1–42)_ did not exert any significant inhibitory effects on this phenotype and was not able to rescue a loss of synapses observed after 24 h of treatment with Aβ_(1–42)_ (Figure 2B,C). However, neurons treated with 100 μM KLVFF, even if not impacting significantly on dendritic fragmentation, showed values closest to the untreated controls and significantly more synapses compared to neurons treated with Aβ_(1–42)_ alone. These data suggest that a high dose of KLVFF (100 μM) could impact the synaptopathy connected to Aβ_(1–42)_ aggregation, which is in line with the observed trend (even if not significantly relevant) in the reduction of the area of Aβ plaques and the increase in the number of aggregates.

Disaggregation tests were performed by exposing neurons to Aβ_(1–42)_ for 24 h and subsequent administration of KLVFF peptides (10 μM or 100 μM) and further incubation for 24 h. We then analyzed the effect on aggregates, dendrite fragmentation, and synapses (Figure 2E–H). In detail, the addition of 10 μM KLVFF peptides was not able to produce any kind of effect, on area or on number of aggregates (Figure 2E), while increasing the dose of KLVFF up to 100 μM led to a significant reduction in plaque areas and correspondingly to an increase in the number of aggregates (Figure 2E). This result is strictly coherent with a disaggregation process of plaques, which is in constant equilibrium with the aggregation process [24,25,26,27]; therefore, we can hypothesize that the action of KLVFF could be related to an interference with these processes, meaning both an action on disaggregation and inhibition of re-aggregation, which could result in an increase in smaller aggregates. Considering the effect on dendrites and synaptopathy, after 24 h, Aβ_(1–42)_ significantly led to an increase in the number of fragmented dendrites labeled by MAP2 signals, (Figure 2F,H) and a significant loss of synapses (Figure 2G,H) compared to untreated control (red dashed line). On the contrary, the treatment with 100 μM KLVFF peptide was able to completely prevent the Aβ_(1–42)_ induced pathology in vitro, as exemplified by a significant decrease in dendrite fragmentation and increase in synaptic levels (Figure 2F,G). Exemplary images are shown in (Figure 2H).

Therefore, we hypothesize that free KLVFF peptides are able to interact with Aβ pathology depending on both the dose (10 or 100 mM) and protocol regimen (concurrently with Aβ_(1–42)_ or after incubation with Aβ_(1–42)_), with a particular emphasis on the identification of the most successful dose (100 μM KLVFF) which is necessary to elicit clear and significant effects on AD pathology in this model system.

### 3.3. NPs Delivering an Equivalent of 100 μM KLVFF Peptides are Non-Toxic for Neurons

**Figure 3:**
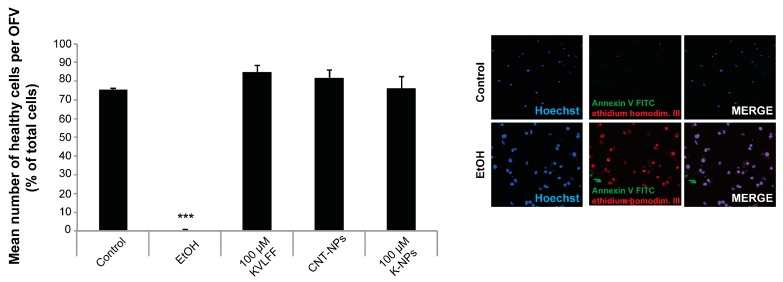
NPs delivering an equivalent of 100 μM KLVFF peptides are non-toxic for neurons. Toxicity profile of ethanol (positive control), CNT-NPs, 100 μM free KLVFF peptide, and NPs delivering 100 μM KLVFF peptide. Neuronal cells were treated for 24 h and compared to untreated control. Apoptotic cells were identified using Annexin V labeled with FITC (Fluorescein isothiocyanate), necrotic cells by ethidium homodimer III, and the total number of cells was assessed using Hoechst 33342, labeling all nuclei. A total of three optic fields of view per condition was analyzed. No significant effect of any treatment except ethanol was found for neuronal cell health (one-way ANOVA followed by Tukey test). Exemplary images for Control and positive Control (EtOH) is shown in the right panel.

Given that 100 μM KLVFF peptide is needed to create an in vitro effect, our next aim was to exclude any possible toxicity of treatment with NPs compared to the free peptide. Therefore, CNT-NPs or K-NPs in such quantities were tested to evaluate possible impacts on cell health. Neurons were incubated with an number of NPs delivering 100 μM KLVFF, or an equal number of CNT-NPs (Figure 3), and cell health was assessed by measuring the number of apoptotic and necrotic cells (together counted as dead cells) after 24 h of exposure and compared to untreated controls (negative control). A treatment with 70% EtOH was included as a positive control (able to produce toxic effects). The results showed no significant difference between untreated cells and those treated with either 100 μM free KLVFF, unloaded NPs (CNT-NPs), or KLVFF-loaded NPs (K-NPs), with the only toxicity arising from the 70% ETOH positive control.

### 3.4. NPs Delivering an Equivalent of 100 μM KLVFF Peptides Reduce Aβ_(1–42)_ Aggregation In Vitro

**Figure 4:**
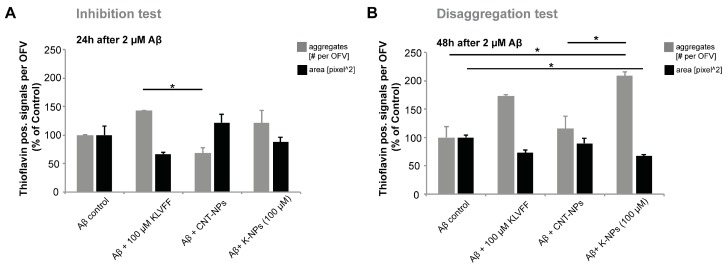
NPs delivering an equivalent of 100 μM KLVFF peptides reduce Aβ_(1–42)_ aggregation in vitro. (**A**,**B**) Hippocampal neurons were treated for either 24 h (**A**) or 48 h (**B**) with 2 μM Aβ_(1–42)_, and the number and area of Aβ aggregates were visualized by Thioflavin staining and measured in three optic fields of view (OFV) 24 h after addition of 100 μM KLVFF peptides, CNT- NPs, or NPs delivering the equivalent of 100 μM KLVFF. (**A**) Regarding the area, no significant changes were observed after 24 h treatment (one-way ANOVA, *p* = 0.115). Regarding the number of Aβ aggregates per OFV, a significant difference was found (one-way ANOVA, *p* = 0.044). Tukey post hoc analysis revealed a significantly higher number of aggregates in cells treated with 100 μM KLVFF peptides compared to cells treated with CNT-NPs (*p* = 0.038). (**B**) After 48 h exposure to Aβ_(1–42)_, regarding the area, significant changes were observed (one-way ANOVA, *p* = 0.042). Tukey post hoc analysis revealed a significant decrease in the area of aggregates in cells treated with NPs delivering 100 μM KLVFF peptide compared to cells treated with Aβ_(1–42)_ only (*p* = 0.046). Regarding the number of Aβ aggregates per OFV, a significant difference was found (one-way ANOVA, *p* = 0.002). Tukey post hoc analysis revealed a significantly higher number of aggregates in cells treated with NPs delivering 100 μM KLVFF peptide compared to cells treated with Aβ_(1–42)_ only (*p* = 0.023), and a significantly higher number of aggregates in cells treated with NPs delivering 100 μM KLVFF peptide compared to cells treated Aβ_(1–42)_ and CNT-NPs (*p* = 0.038).

Next, we replicated the same types of experiments (inhibition versus disaggregation) to investigate further whether KLVFF delivered by NPs shows similar effects on AD pathology as the free KLVFF peptide reported before. To that end, as in the inhibition tests before, rat hippocampal neurons were treated simultaneously with 2 μM Aβ_(1–42)_ peptide and 100 μM free KLVFF peptide, CNT-NPs, or K-NPs delivering the equivalent of 100 μM KLVFF peptide and incubated for 24 h. Disaggregation tests were performed as shown before, namely 24 h of incubation with Aβ_(1–42)_ and then further 24 h incubation after the addition of 100 μM free KLVFF peptide, CNT-NPs, or K-NPs delivering the equivalent of 100 μM KLVFF peptide. Aβ_(1–42)_ aggregates were visualized using Thioflavin and the number per optic field of view and their mean area measured (Figure 4). In the inhibition test (Figure 4A), even if, as shown in the previous experiment, a trend towards a reduction was visible, independently of the tested samples, no significant changes regarding the area of aggregates were detected for the control. On the contrary, disaggregation tests (Figure 4B) showed relevant changes in the area of Aβ aggregates, namely a significant and more substantial decrease in the area of aggregates in cells treated with NPs delivering 100 μM KLVFF peptide compared to other samples, in particular, free KLVFF. Simultaneously and coherently with the decrease in the area of aggregates, the number of aggregates was significantly increased in cells treated with NPs delivering 100 μM KLVFF peptide (Figure 4B) compared to cells subjected to the other treatments (control cells incubated with Aβ_(1–42)_ only, CNT-NP administration, and free KLVFF peptide). Therefore, exposure to KLVFF peptides may impact Aβ aggregation, especially on the disaggregation/re-organization of aggregates, shifting the profile of aggregates from large complexes into more but smaller aggregates. Furthermore, KLVFF peptide delivery by NPs achieved an advantage over free KLVFF peptides, probably due to increased peptide stability.

### 3.5. NPs Delivering an Equivalent of 100 μM KLVFF Peptides Reduce Aβ_(1–42)_ Induced Pathology In Vitro

**Figure 5:**
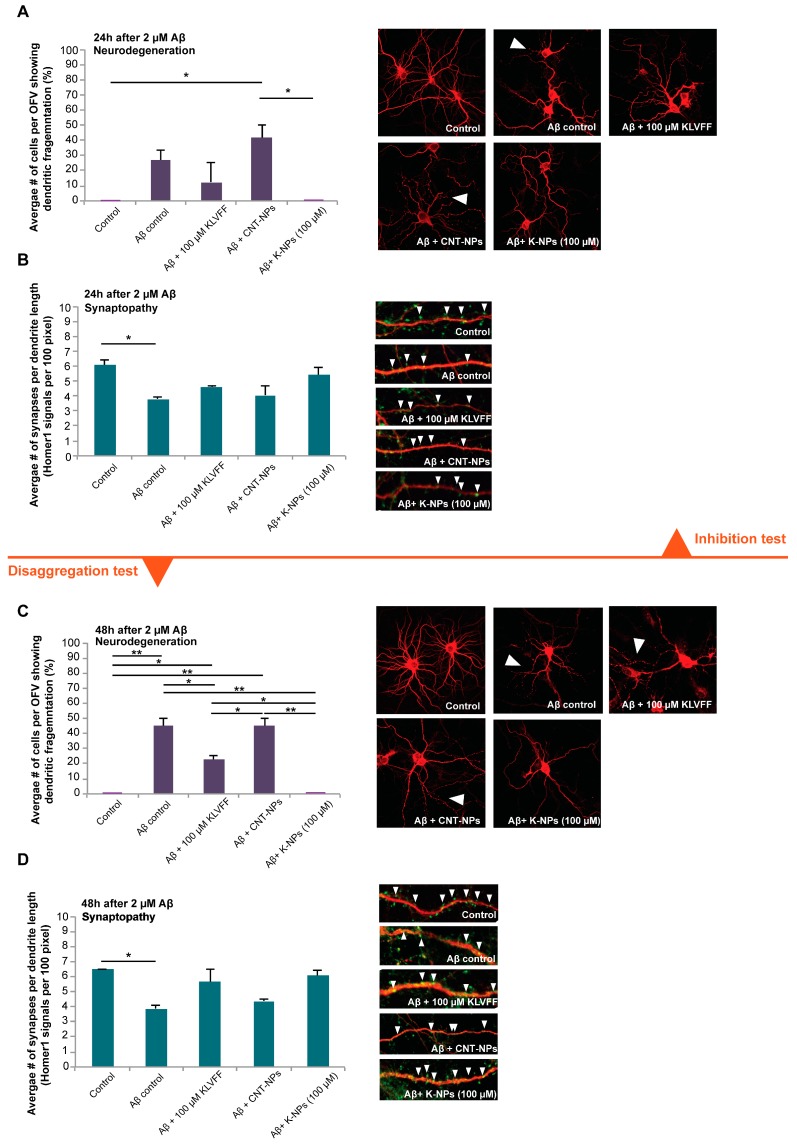
Neurons were treated for either 24 h (**A**,**B**) or 48 h (**C**,**D**) with 2 μM Aβ_(1–42)_, and dendritic fragmentation (**A**,**C**) and synapse density (**B**,**D**) were evaluated by MAP2 staining and by measuring the number of Homer1 positive signals per dendrite length from three optic fields of view (OFV) with at least 10 cells 24 h after addition of 100 μM KLVFF peptides, CNT-NPs, or NPs delivering the equivalent of 100 μM KLVFF. Images show MAP2 (red) and Homer1 (green). Exemplary synapses are labeled with arrows. A more detailed caption with the relevant statistical analysis data is reported in the supplementary material.

Consequently, we investigated whether the reduction in Aβ_(1–42)_ aggregation had an effect on the pathological changes in neurons induced by Aβ_(1–42)._ To that end, neurons were analyzed to assess whether, after the same treatment as described above, any amelioration in neurodegeneration and synapse loss could be revealed (Figure 5). 

Our data reveal similar results to those obtained from incubation with free KLVFF peptides. In the inhibition studies, we could observe significant differences in terms of the number of cells with dendritic fragmentations after exposure to Aβ_(1–42)_ between cells treated with CNT-NPs and K-NPs, but not between CNT-NPs and cells treated with free peptide (Figure 5A). Only OFVs with control samples (cells without any treatment) and cells with co-administration of Aβ_(1–42)_ and K-NPs showed a complete absence of fragmentation. A significantly lower synapse density was observed in Aβ_(1–42)_ treated cells. No significant differences in synapse density assessed by the number of Homer1 positive immunofluorescent puncta per dendrite length averaged from three dendrites per cell from ten cells per condition were observed for treatment with free peptide or NPs (Figure 5B).

In disaggregation studies (Figure 5C,D), incubation with Aβ_(1–42)_ over 48 h induced significant dendritic fragmentation compared to healthy control cells. The addition of unloaded CNT-NPs (vehicle) did not lead to significant modification of this pathology. The free KLVFF peptide confirmed the ability to significantly reduce the number of cells with dendritic fragmentation. Only the addition of NPs delivering the equivalent of 100 μM KLVFF peptide was able to completely restore cell health, resulting in a significantly lower number of fragmented cells compared to all the controls (except for healthy cells) and also significantly lower numbers compared to free KLVFF peptide (Figure 5C). Synapse density was significantly reduced by treatment with Aβ_(1–42)_ over 48 h. The addition of both the free KLVFF peptide and K-NPs after 24 h prevented this decrease. No significant differences were detected between healthy untreated controls and cells treated with Aβ_(1–42)_ plus 100 μM KLVFF peptide and Aβ_(1–42)_ plus 100 μM K-NPs (Figure 5D). However, Aβ_(1–42)_ plus 100 μM KLVFF peptide and Aβ_(1–42)_ plus 100 μM K-NPs treated cells were also not significantly different from cells treated with Aβ_(1–42)_. Thus, K-NPs were able to partially rescue the Aβ induced synaptopathy with similar efficacy to free KVLFF peptide, but K-NPs were even more efficient in preventing Aβ induced neurodegeneration.

## 4. Discussion

The brain-targeted delivery of active molecules with poor aqueous solubility, low stability, and an inability to cross the BBB is currently a major challenge in the development of novel treatment strategies for neurodegenerative disorders. In particular, recent research on AD has revealed several compounds with high potential to act as therapeutics; however, due to their chemical nature and the thereby existing impairments mentioned above, these potential therapeutic strategies cannot be easily translated to clinical practice. Among the proposed compounds, the KLVFF peptide showed promising results for the treatment of AD. Here, we report the successful development of KLVFF loaded polymeric NPs that have been previously demonstrated to cross the BBB through the addition of a g7 ligand [28].

Recently, we demonstrated the possibility of delivering an active compound across the BBB using g7-NPs injected in a mouse model for AD [29]. This shows a promising approach to assure the protection of the cargo in blood and a controlled release inside the brain using NPs. The KVLFF peptide is another potent candidate for AD treatment. KLVFF has been specifically designed to target a central region of Aβ and was shown to inhibit the elongation to fibrillar aggregates in vitro [5,6]. Here, we confirm the effects of KVLFF on Aβ aggregation at a concentration of 100 μM and in the presence of 2 μM Aβ. We observed stronger effects on existing larger aggregates (disaggregation) as on the prevention of aggregation (inhibition). However, the application of a broader range of concentrations and variations in exposure times in the future may identify conditions in which the efficacy of the peptide is further improved. The presence of KVLFF seems to have complex effects on the dynamics of aggregation and disaggregation of Aβ, and might result in a shift towards more but smaller aggregates with limited potential for growth rather than preventing aggregation altogether. More importantly, 100 μM KVLFF delivered by NPs had similar effects compared to the free peptide. Future studies will need to evaluate whether maintaining Aβ in a state of smaller aggregates or disaggregating plaques into smaller aggregates through the chronic application of the peptide is an effective treatment approach, as not soluble oligomers may be more toxic than larger Aβ aggregates, although in our study, a beneficial effect was observed on the neuropathology in vitro.

While a previous study could show the effects of KVLFF nanocomposites on the viability of PC12 cells after exposure to Aβ [15], we performed a more thorough analysis using primary neurons. In this study, we could observe that free KVLFF and, even more potent, NP delivered KVLFF significantly reduces dendritic fragmentation, a sign of neurodegeneration. Neurodegeneration is a hallmark of AD, and Aβ-induced toxicity was shown to be a major contributor through various mechanisms, such as oxidative stress, triggering inflammatory responses, and the generation of trace metal dyshomeostasis. In addition, the presence of Aβ was shown to have direct effects on synapses [30,31] and zinc-depletion [32]. Here, we could also observe a significant reduction in synapse density after the application of Aβ. This reduction was absent after treatment with free KLVFF peptide and K-NPs. Given that neurodegeneration and synapse loss may be the best correlate to the observed cognitive decline in AD, K-NPs being able to deliver KLVFF into the brain may be a promising approach. In particular, due to the results obtained, if confirmed in AD animal models, the efficacy of the KLVFF peptide could be exploited in the treatment of late-stage AD and not only in the starting steps of the disease, opening up treatment to a more relevant number of patients to be managed.

## 5. Conclusions

Taken together, we have provided a method for the generation of KVLFF peptide-loaded NPs and have shown that these K-NPs have equal or increased activity compared to the free peptide, possibly due to increased peptide stability. Thus, these K-NPs possess full therapeutic potential and overcome the limitations for brain-targeted delivery of the free peptide.

## Supplementary Materials

The following are available online at https://www.mdpi.com/1999-4923/11/11/572/s1, Table S1: Solubility of KLVFF, PLGA, and mix of KLVFF and PLGA in water and suitable organic solvent for nanoprecipitation techniques. Figure S1: Characterization of Aβ.
