# Supplementary material for: Nanomedicine against Aβ Aggregation by β–Sheet Breaker Peptide Delivery: In Vitro Evidence
Source: Pharmaceutics. 2019 Nov 1;11(11):572. doi: 10.3390/pharmaceutics11110572 (PMC6920811; doi:10.3390/pharmaceutics11110572)
Supplement: Supplementary file 1 [file pharmaceutics-11-00572-s001.pdf]

pharmaceutics

# Nanomedicine Against Aβ Aggregation by β–sheet Breaker Peptide Delivery: In Vitro Evidence

**Francesca Pederzoli, Barbara Ruozi, Jason Duskey, Simone Hagmeyer, Ann Katrin Sauer, Stefanie Grabrucker, Romina Coelho, Natalia Oddone, Ilaria Ottonelli, Eleonora Daini, Michele Zoli, Maria Angela Vandelli, Giovanni Tosi and Andreas M. Grabrucker**

**Table 1.** solubility of KLVFF, PLGA and mix of KLVFF and PLGA in water and suitable organic solvent for nanoprecipitation techniques.

| Solvent | KLVFF Solubility | PLGA RG-503H Solubility | KLVFF/PLGA-RG-503H Solubility |
|---|---|---|---|
| Water | Insoluble | Insoluble | Insoluble |
| PVA 1% *w/v* water solution | Soluble (1.0 mg/mL) | Insoluble | Insoluble |
| DMSO | Very soluble (> 10 mg/mL) | Very soluble (> 10 mg/mL) | 1 mg of KLVFF +10 mg of PLGA/mL |
| Acetone | Insoluble at r.t. Soluble at 50°C (0.10 mg/mL) | Very soluble at r.t. (> 20 mg/mL) | 200 uL of 3.75 mg/mL KLVFF DMSO solution in 12.5 mg/mL PLGA hot acetone solution under stirring |
| Methanol | Slightly soluble at r.t. Soluble at 55°C (1.80 mg/mL) | Soluble at 50°C (hot solution) (15 mg/mL) | 1.25 mg/mL KLVFF hot methanol solution in 12.5 mg/mL PLGA hot acetone solution under stirring |

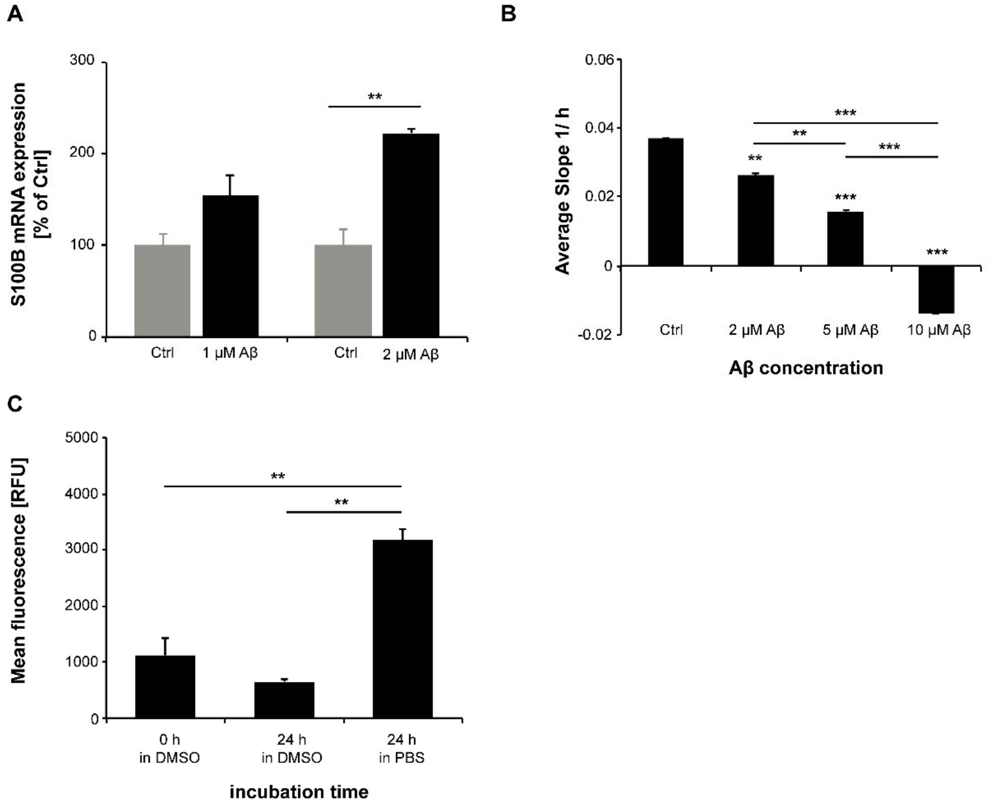


**Figure S1.** Charaterization of Aβ used in the study. **A,B**) DI TNC1 astrocytes were grown for 24 h and incubated for 48 h with different concentrations of $A\beta_{(1-42)}$. **A)** Expression levels of S100B, a cytokine reported to be upregulated in presence of Aβ in vitro and in vivo was measured using qRT-PCR.

While application of 1 µM Aβ$_{(1–42)}$ does not result in significant upregulation compared to Controls, 2 µM Aβ$_{(1–42)}$ significantly induced S100B gene expression (t-test, $p = 0.0097$). **B**) Cell health was assessed measuring impedance using an xCelligence RTCA system. Cells were grown for 24 h before treatment and monitored for further 36 h after treatment. Application of 2 µM also leads to a significant decrease in cell health over time (one-way ANOVA followed by Tukey test, Ctrl vs 2 µM Aβ$_{(1–42)}$: $p = 0.0012$, Ctrl vs 5 µM Aβ$_{(1–42)}$: $p = 0.0002$, Ctrl vs 10 µM Aβ$_{(1–42)}$: $p < 0.0001$). **C**) Thioflavin T (ThT) aggregation assay: 2.5 µg Aβ dissolved in DMSO were added to 50 mM Tris buffer pH 7.4 containing 1 mg/ml ThT. The relative ThT fluorescence intensity was measured immediately after adding Aβ, and 24 h after addition to investigate Aβ aggregation. ThT buffer without Aβ was used as blank. The results show initially very low fluorescence indicating Aβ to be mostly monomeric. Dissolved in DMSO, Aβ remains mostly monomer after incubation for 24 h at RT due to the presence of DMSO. Dilution of DMSO with PBS (DMSO:PBS 1:3) induces significant Aβ aggregation after incubation for 24 h at RT (one-way ANOVA ($p = 0.0004$) followed by Tukey test, DMSO 0 h vs 24 h: $p = 0.294$, DMSO 0 h vs PBS 24 h: $p = 0.00163$, DMSO 24 h vs PBS 24 h: $p = 0.00101$).

Figure 2. KLVFF peptides show beneficial effects in an in vitro model for AD. Hippocampal neurons were treated for either 24 hours with2 µM Aβ$_{(1–42)}$ together with KVLFF (left panels: Inhibition test) or 48 hours with 2 µM Aβ$_{(1–42)}$ with KVLFF added after 24 h (right panels: Disaggregation test). A,E) The number and area [in pixel$^2$] of aggregates was visualized by Thioflavin staining and measured in three optic fields of view (OFV). The results are shown in % of Control. A) In the inhibition test, the results show a trend towards an increase in number of aggregates. A significantly lower average size of aggregates was detected in neurons treated with 100 µM KLVFF compared to those treated with 10 µM KLVFF (one-way ANOVA, $p = 0.049$, followed by Tukey test, $p = 0.043$) but not compared to Aβ$_{(1–42)}$ treated controls. B,F) The number of cells showing fragmented dendrites (MAP2 staining) was analyzed. Further, (C,G), a "synaptopathy" was evaluated by measuring the number of Homer1 positive signals per dendrite length. Three optic fields of view (OFV) with at least 10 cells were evaluated. B) For the inhibition test, the results show a trend towards a rescue of neurodegeneration with 100 µM KLVFF but no significant overall differences were detected (one-way ANOVA). C) A significant difference was detected in synapse density between neurons treated with 100 µM KLVFF compared to those treated with Aβ$_{(1–42)}$ (one-way ANOVA followed by Tukey test, $p = 0.0238$). D) Left panel: MAP2 staining showing dendritic fragmentation as early sign of neurodegeneration. Right panel: MAP2 (green) and Homer1 (red) staining visualizing synapses along a dendrite. E) In the disaggregation test, after 48 h, a significantly lower average size of aggregates was detected in neurons treated with 100 µM KLVFF compared to Aβ$_{(1–42)}$ treated controls and those treated with 10 µM KLVFF (one-way ANOVA, p = 0.016 followed by Tukey test, Aβ$_{\text{Control}}$ vs Aβ + 100 µM KLVFF, $p = 0.026$; Aβ + 10 µM KLVFF vs Aβ + 100 µM KLVFF, $p = 0.023$). F) 48 h after treatment, a significant increase in neurons showing fragmented dendrites compared to untreated controls was detected in neurons treated with Aβ$_{(1–42)}$ and Aβ$_{(1–42)}$ plus 10 µM KLVFF (one-way ANOVA followed by Tukey test). Neurons treated with Aβ$_{(1–42)}$ plus 100 µM KLVFF did not show a significant difference to untreated controls and had significantly less fragmented dendrites compared to those treated with Aβ$_{(1–42)}$ and Aβ$_{(1–42)}$ plus 10 µM KLVFF (one-way ANOVA followed by Tukey test, Aβ$_{(1–42)}$ vs Aβ$_{(1–42)}$ plus 100 µM KLVFF: $p = 0.0002$, Aβ$_{(1–42)}$ plus 10 µM KLVFF vs Aβ$_{(1–42)}$ plus 100 µM KLVFF: $p = 0.0019$). G) A significantly lower synapse density was found in neurons after 48 h exposure to Aβ$_{(1–42)}$. This decrease was prevented by treatment with 100 µM KLVFF after 24 h for 24 h (one-way ANOVA followed by Tukey test: Aβ$_{(1–42)}$ vs Aβ$_{(1–42)}$ plus 100 µM KLVFF: $p = 0.0096$). H) Left panel: MAP2 staining showing dendritic fragmentation as early sign of neurodegeneration. Right panel: MAP2 (green) and Homer1 (red) staining visualizing synapses along a dendrite.

Figure 5. NPs delivering an equivalent of 100 µM KLVFF peptides reduce Aβ$_{(1–42)}$ induced pathology in vitro. Neurons were treated for either 24 hours (A) or 48 hours (B) with 2 µM Aβ$_{(1–42)}$, and dendritic fragmentation and synapse density were evaluated by MAP2 staining and by measuring the number of Homer1 positive signals per dendrite length from three optic fields of view (OFV) with at least 10 cells 24 h after addition of 100 µM KLVFF peptides, CNT-NPs, or NPs delivering the equivalent of 100 µM KLVFF. A) 24 h after treatment with Aβ$_{(1–42)}$, significant

differences in the number of cells with dendritic fragmentations (arrows) were observed (one-way ANOVA, $p = 0.0282$). While untreated controls and cells treated with $A\beta_{(1–42)}$ and 100 µM KLVFF did not show cells with dendritic fragmentations, some cells were found in $A\beta_{(1–42)}$, $A\beta_{(1–42)}$ and free KLVFF peptide, and $A\beta_{(1–42)}$ and CNT-NP treated cells. A significant increase in dendritic fragmentations after exposure to $A\beta_{(1–42)}$ was observed in cells treated with empty CNT-NPs compared to untreated controls (Tukey post hoc test, $p = 0.041$). Treatment with K-NPs but not free peptide significantly reduced dendritic fragmentations compared to CNT-NP treated cells (Tukey post hoc test, $p = 0.0411$). B) A significant loss in synapse density was observed (one-way ANOVA, p = 0.0421, Tukey post hoc test: Control vs. $A\beta_{(1–42)}$: $p = 0.0491$). All other conditions were not significantly different from controls. Images show MAP2 (red) and Homer1 (green). Exemplary synapses are labeled with arrows. C) 48 h after treatment with $A\beta_{(1–42)}$, significant differences in the number of cells with dendritic fragmentations were observed (one-way ANOVA, p = 0.0004). Treatment with $A\beta_{(1–42)}$ induced a significant increase in cells with dendritic fragmentation compared to untreated controls after 48 h ($p = 0.001$). The addition of CNT-NPs did not lead to significant modification of this pathology and similarly to $A\beta_{(1–42)}$ only treated cells, a significant increase in cells with dendritic fragmentation compared to untreated controls was seen ($p = 0.001$). Addition of free KLVFF peptide significantly reduced the amount of cells with dendritic fragmentation compared to $A\beta_{(1–42)}$ ($p = 0.026$) and $A\beta_{(1–42)}$ plus NPs ($p = 0.026$), but still results in a significant increase in fragmentation compared to untreated controls ($p = 0.026$). The addition of NPs releasing the equivalent of 100 µM KLVFF peptide completely restored cell health resulting in no significant difference to untreated controls, but a significantly lower number of fragmented cells compared to $A\beta_{(1–42)}$ ($p = 0.0012$), $A\beta_{(1–42)}$ plus free KLVFF peptide ($p = 0.02606$), and $A\beta_{(1–42)}$ plus NPs ($p = 0.026$). D) Synapse density was significantly altered after 48 h (one way ANOVA, $p = 0.025$). The addition of $A\beta_{(1–42)}$ caused a significant decrease in synapse density compared to untreated controls ($p = 0.032$). The addition of free KLVFF or NP delivered KLVFF prevented a significant loss of synapses.

© 2019 by the authors. Licensee MDPI, Basel, Switzerland. This article is an open access article distributed under the terms and conditions of the Creative Commons Attribution (CC BY) license (http://creativecommons.org/licenses/by/4.0/).
